# The Role of Tobacco-Derived Carcinogens in Pancreas Cancer

**DOI:** 10.5402/2011/249235

**Published:** 2011-07-17

**Authors:** Rajiv Lochan, Helen L. Reeves, Anne K. Daly, Richard M. Charnley

**Affiliations:** ^1^Hepato-Pancreato-Biliary Unit, Department of Surgery, Freeman Hospital, Newcastle upon Tyne NE7 7DN, UK; ^2^Northern Institute for Cancer Research, Newcastle University, Newcastle upon Tyne NE2 4HH, UK; ^3^Institute of Cellular Medicine, Newcastle University, Newcastle upon Tyne NE2 4HH, UK

## Abstract

The extremely poor outcome from pancreas cancer is well known. However, its aetiology less well appreciated, and the molecular mechanisms underlying this are poorly understood. Tobacco usage is one of the strongest risk factors for this disease, and this is a completely avoidable hazard. In addition, there are well described hereditary diseases which predispose, and familial pancreas cancer. We have sought here to summarise the role of tobacco-derived carcinogens and the mode of their tumorigenic action on the pancreas. There is compelling evidence from animal and human studies (laboratory including cell line studies and epidemiologic) that tobacco derived carcinogens cause pancreas cancer. However, the manner in which they do so is not entirely apparent. There is also compelling evidence that synergism with genetic and other life-style factors—like diet obesity—results in a multifactorial causation of the disease. Ascertaining the role of tobacco carcinogens in the development of this cancer and their interaction with other risk factors will enable novel therapeutic and preventative strategies to improve outcome from this appalling malignancy.

## 1. Introduction

Adenocarcinoma of the pancreas is a devastating disease, and only a small proportion of sufferers achieve an attempt at curative surgical treatment which leads to a median survival of about 12 months [1]. In spite of advances in surgical technology, perioperative care, and adjuvant treatments the improvement in outcome from this disease has been minimal. Improving survival could be the result of an understanding of the biology of the cancer, and its risk factors with a view to detecting it early—possibly in high-risk individuals with secondary prevention strategies in selected patients and also devising novel modes of interfering with its initiation and progression. 

There have been numerous lines of evidence implicating tobacco smoking as a risk factor for pancreatic cancer and this has been well recognised in the field of pancreatology. Realisation of this in the wider healthcare community, however, had been limited until the publication of the 2004 US Surgeon General's report on health consequences of Smoking [2]. Tobacco usage is the single largest preventable cause of disability disease and death in the developed world and probably also plays a great role in morbidity and mortality amongst people within the developing world. 

A recent meta-analysis [3] demonstrated the significant strength of the association between cigarette smoking and pancreas cancer and calculated that the population attributable risk secondary to tobacco use for the malignancy was about 20%. Genetic factors play a role in about 10% of incident cases. However, the exact etiological cause for the vast majority of these cancers continues to remain speculative. 

Tobacco smoking is the strongest risk factor for pancreas cancer, but the mechanism of disease causation has not been elucidated although the various steps in the sequence of development of the malignancy have been described—beginning from PAN-IN 1 to invasive adenocarcinoma [4]. On a background of this recent meta-analysis summarising the evidence linking tobacco use with pancreatic cancer, we have reviewed the available laboratory and experimental data, providing a summary of the current knowledge of tobacco-driven pancreas carcinogenesis.

## 2. Search Methods

Established medical databases including PUBMED, MEDLINE, SCOPUS, and INDEX MEDICUS were searched for a variety of different combination of the terms *pancreas, pancreatic, cancer, adenocarcinoma, tobacco, use, smoking, and carcinogens *to identify peer-reviewed publications exploring the relationship between tobacco smoking and pancreas cancer. After excluding duplicate results, a total of 235 publications were initially identified. They were subjected to careful scrutiny and we excluded case-control, cohort studies and other cross-sectional/longitudinal studies published up until 2008. This was due to an excellent and comprehensive meta-analysis summarising the epidemiology of tobacco use and pancreas cancer (vide supra) published in 2008. All publications dedicated to laboratory, experimental and clinical consequences of tobacco carcinogen effect on the pancreas or development of pancreas cancer were identified and the full-text was carefully scrutinised. We also examined epidemiological papers published after 2008. Thus for the purposes of this paper 115 publications were reviewed in detail.

## 3. Review

Nicotine is the major psychoactive substance in tobacco and is responsible for tobacco dependence and addiction. It is thought to induce a euphoric state in users by the activation of the mesolimbic dopaminergic reward system in the nucleus accumbens of the brain [5], and this may be related to the highly addictive properties of the drug [6]. 

Individuals have genetically based differences in their ability to metabolize nicotine as well as genetic differences in the psychological reward pathways that may influence individual response to smoking initiation, dependence, addiction and cessation. While nicotine itself has some cancer-promoting abilities which we will discuss later, most of the health consequences of tobacco use are secondary to the other chemicals within it. Some are nicotine metabolites, whilst the others are unrelated.

### 3.1. Tobacco-Derived Carcinogens

Tobacco smoke contains 4000 compounds and 50 carcinogens as compared to 3000 compounds and 30 carcinogens in processed unburnt tobacco [7]. Both mainstream and sidestream smoke are harmful and have differing proportions of carcinogens. These carcinogens include polynuclear aromatic hydrocarbons (PAH), tobacco-specific nitrosamines (TSNA), aromatic amines, aza-arenes, and aldehydes, other organic compounds like benzene, inorganic compounds like hydrazine, and various metals [8] (reviewed by Hecht and Hoffmann). Seven compounds have been identified in the family of TSNA's—NNN (*N*′-nitrosonornicotine), NNK (4-(methylnitrosamino)-1-(3-pyridyl)-1-butanone), NNAL (4-(methylnitrosamino)-1-(3-pyridyl)-1-butanol), NAT (*N*′-nitrosoanatabine), NAB (*N*′-nitrosoanabasine), iso-NNAL [iso 4-(methylnitrosamino)-4-(3-pyridyl)-1-butanol] and iso-NNAC [iso 4-(*N*-methylnitrosamino)-4-(3-pyridyl)butyric acid)]. NNN, NNK, and NNAL are the most powerful carcinogens. Nitrosation of nicotine during tobacco processing and cigarette smoking leads to the formation of tobacco-specific nitrosamines, of which 4-(methylnitrosamino)-1-(3-pyridyl)-1-butanone (NNK) [9] is the most notorious and is now classified by the International Agency for Research on Cancer as a Group 1 carcinogen. In the context of pancreatic cancer TSNAs, NNK and NNAL are particularly important, as they demonstrate organ specificity towards the pancreas. They are present in large quantities in both burnt and unburnt tobacco. 

TSNAs are procarcinogens. Following exposure, they need to undergo a series of steps uptake, metabolic activation, and DNA and protein adduct formation which subsequently leads to altered growth kinetics in the target organ and development of neoplasia. These metabolic pathways of NNK and NNN have been demonstrated *in vivo *in mice [10], rats, and hamsters [11] and in subcellular fractions, cultured cells and tissues from animals [12] and humans [13].

NNK can undergo hydroxylation, oxidation and carbonyl reduction to yield numerous metabolites which include diazoxides, formaldehyde, NNK-N-oxide, and NNAL. These metabolites react with DNA and protein resulting in adducts formation. In mice and rats, NNK-derived electrophiles react with nucleophilic centers in DNA to yield a variety of products including 0^6^-methylguanine which causes miscoding of DNA during replication [14]. This production of 0^6^-methylguanine and 0^6^-ethylguanine had also been confirmed histochemically in pancreatic duct cells in vitro [15].

Until recently, nicotine had been considered to be responsible for only the addictive property of tobacco only; however, of late it is being increasingly recognised that nicotine might independently possess cancer facilitating properties causing the metastasis of tumours preinitiated by tobacco carcinogens [16] in rat models of lung cancer. This effect is thought to be mediated through nicotine stimulation of nAChR (nicotinic acid acetyl choline receptors). Nicotine can also induce epithelial-mesenchymal transition in cultured lung, breast and pancreatic cancer cells [17]. This is an essential part of the invasion—metastasis sequence and therefore it appears that while nicotine has only limited capacity to initiate tumour formation, it can induce invasion and metastasis in preexistent tumours.

### 3.2. Tobacco-Derived Carcinogens and Pancreatic Tumours in Animals

Numerous animal models of pancreas ductal cancer have been developed, and these have proved invaluable in elucidating the kinetics of the carcinogen within physiological systems and pathogenesis of ductal cancer of the pancreas. In 1988, the important role of tobacco derived carcinogens in extrapulmonary cancers including the pancreas was highlighted in a commentary by Hecht and Hoffmann. The same commentary also concluded that two of the nicotine-derived nitrosamines, NNK and NNN, are strong carcinogens in laboratory animals and that they could induce tumors both locally and systemically. Also, the magnitude to the total doses of NNK and NNN required to produce cancer in laboratory animals is similar to the total estimated doses which long-term snuff-dippers or heavy smokers are exposed life time [8].

In F-344 rats, NNK and NNAL cause development of not only lung but also pancreatic adenocarcinoma. The doses of carcinogens to body weight needed to cause these neoplasms in rats were similar to lifetime exposure of these chemicals in heavy smokers (40 cigarettes/day). This was the first example of pancreatic tumour induction by a constituent of tobacco smoke. NNAL appeared to be the proximate pancreatic carcinogen of NNK as it induced more tumours than NNK [18]. They are the only carcinogens to induce pancreatic adenocarcinoma in animal models when given systemically [18, 19]. Also, in F-344 rats, there is evidence that following oral NNK administration, there is preferential metabolism of NNK to (S)-NNAL followed by its extensive retention in various target tissues including the pancreas of NNK-orally treated animals [20]. Treatment of rats with TSNA's also resulted in the induction of pancreatic acinar cell and ductal cell neoplasms [21].

In rodents it has been shown that NNK and NNAL are excreted in the bile in significant concentrations [22]. If this is true in humans, it may be the route through which activated carcinogens reach the head of pancreas (carcinogen containing bile refluxing into the pancreatic duct). This theory is attractive given that the pancreatic head is the most frequent site for adenocarcinoma [23–25]. A study in rhesus monkeys (*n* = 4), however, found that biliary excretion of the NNK metabolites was significantly less than predicted from rat experiments. Very limited experiments have been performed on this route of TSNA excretion.

### 3.3. Tobacco Carcinogens in Humans and Their Relevance to Pancreatic Cancer

Most animal studies have been extrapolated to humans and the assumption is that the metabolism and physiological distribution kinetics of tobacco derived carcinogens is similar to that in rats and rodents. However, there will be differences in the manner in which our species handles these carcinogens, and a few studies have tried to elucidate this by various means. Essentially, what has been achieved is to show that the pancreas is exposed to these chemicals and that these metabolites bind to pancreatic ductal cell DNA and result in mutations there and that various phenotypic changes occur there including cancer. However the exact mechanism by which these carcinogens turn a normal ductal cell into a malignant one has not been described. 

TSNA's (NNK and NNAL) have been detected in the pancreatic juice of smokers in significantly higher quantities as compared to nonsmokers confirming that the pancreas is exposed to these carcinogens [26]. It has been shown that aromatic amines and nitroaromatic hydrocarbons are metabolically activated in the human pancreas [27]. This confirmed that the pancreas is exposed to TSNA's and that these carcinogens may play a role in carcinogenesis at this site [22].

Although a strong correlation had been suggested between cigarette smoking and pancreatic cancer, studies on pathological changes in the pancreas of smokers are infrequent. Tomioka et al. performed a comparative autopsy study on 73 pancreases obtained by autopsy from 42 heavy cigarette smokers and 31 nonsmoker patients. Although the incidence of pancreatic cancer in smokers was higher than in nonsmokers, the difference was statistically not significant. Ductal changes, including mucinous or squamous cell metaplasia and papillary hyperplasia, were found with equal frequencies in both groups of patients and the authors concluded that the type and the incidence of these ductal alterations were not related to smoking but to the age. There were significant limitations of this autopsy study including limited number of the sections of the pancreas examined, as well as exclusion of other important variables, such as alcohol, diet and diabetes weaken the value of this study [28]. Another autopsy study obtained purified DNA from human lung, liver, bladder, pancreas, breast and cervix of 13 men and 6 women and analysed it for DNA adducts using the nuclease P1 modification of the 32P postlabelling technique. Relatives were asked to provide information on smoking history for deceased subjects. All tissues examined except the breast had detectable adducts. In lung, bladder and pancreatic tissue a characteristic pattern of adducts was seen which had previously been reported as typical of cigarette-smoke-induced damage; diagonal reactive zone [29]. Smokers and former smokers tended to have higher adduct levels than nonsmokers in the tissues examined but this was only significant for the lung. These results confirmed the finding that cigarette smoking is associated with DNA damage in the lung and suggested that similar damage may be related to tobacco-induced neoplasms of other tissues [30]. TSNA's adducts have been found in the pancreas and the levels have correlated with dose and time related to exposure [31].

### 3.4. Mechanism of Tobacco-Induced Carcinogenesis in the Pancreas

Over the past 10–15 years extensive work into the development of pancreatic cancer has been carried out. A model of stepwise progression from normal to malignant cells, and the molecular alterations involved in these has been described [32].

Very similar to the adenoma-carcinoma sequence in the colon, there is neoplastic progression in the pancreatic ducts [33–36]. This progression from a normal pancreatic ductal cell to an infiltrating carcinoma involves sequential multiple genetic alterations. The genetic changes include activating point mutations in K-ras, overexpression of HER-2neu, and inactivation of p16, p53 DPC4, and BRCA2. A gate-keeper gene for the initiation of pancreatic neoplasia has not been indentified and the cause of the ignition of change in a normal ductal cell towards neoplasia is under investigation. 

Several mechanisms have been proposed in regard to how tobacco smoking might be involved in pancreatic carcinogenesis such as tobacco-carcinogen-induced adducts resulting in the mutation of the K-ras oncogene and initiating tumorigenesis and subsequent promotion occurring by other factors,. However, this is not yet clear, and other mechanisms such as the autonomic nervous system and its various pathways, lipid peroxidation, and synergistic molecular damage by other environmental, occupational, and other factors may play an important role.

#### 3.4.1. K-ras Mutations, DNA Adducts, and Tobacco in the Initiation of Pancreatic Cancer

K-ras mutations have been identified in various malignancies, including pancreas cancers. A number of animal studies have been performed examining the hypothesis that tobacco smoking-induced K-ras mutations and that these were the beginnings of the malignancy [15, 37, 38]. In one of these models, an in vitro transformation of immortal hamster pancreatic duct cells was studied after exposure to NNK for varying lengths of time. Analysis of pancreatic DNA for K-ras mutation at codons 12, 13, and 61 showed G–A transition at codon 12 of the K-ras oncogene in tumour cells after 1 and 3 days of NNK treatment. However, no mutation was detected in tumour cells developing 5 and 7 days after NNK treatment [15]. One and 3 day NNK-treated cells were able to grow in the absence of growth factors and serum immediately after the treatment. Also, the tumorigenicity of these transformed cells was determined in nude mice, and the cells treated for 1 and 3 days produced well-differentiated neoplasms, while those that were treated for longer durations did not. Thus, it appeared that although K-ras mutation at codon 12 of pancreatic adenocarcinoma was an early event, it was not necessarily required for the development and/or progression of the tumours in nude mice. 

Numerous studies have been performed in humans to ascertain the role of K-ras in pancreas development with conflicting results. Two key studies reporting on this issue and had divergent results—Nagata et al. observed a negative association [39], whilst Hruban et al. found a positive association [40]. In a population-based case-control study, where information on smoking and other life-style factors was obtained at direct patient interview, no significant association between K-ras mutation and smoking was identified [41]. A meta-analysis of 8 studies investigating K-ras mutations in various cancers including pancreas adenocarcinoma [42], explored the hypothesised association lifetime history of tobacco consumption. No significant association between the frequency of K-ras mutations (OR = 1.26; 95%  CI = 0.82–1.94) and tobacco usage was identified. Subsequently, the PANKRAS 2 study group authors reported a case-case study [43], where they did not find any association between acquisition of K-ras mutations and tobacco smoking. In combination, these studies have contributed to the conclusion that while both smoking and K-Ras mutations are important in the development of pancreas cancer, they are independent of one another.

#### 3.4.2. Promotion

Recent studies have demonstrated over-expression of cyclooxygenase and 5-lipoxygenase receptors in exocrine pancreatic carcinomas [44]. Following on from this, Weddle et al. demonstrated high basal levels of arachidonic acid release and expression of m-RNA for *β* adrenergic receptors (1 and 2) in two human cell lines derived from exocrine ductal pancreatic carcinomas. Notably, exogenous NNK promoted DNA synthesis in these cells [45]. This study therefore underlined the importance of *β*-adrenergic mechanisms in pancreas carcinogenesis (as had previously been reported for lung cancer [46, 47].

NNK reportedly binds with high affinity to adrenergic and nicotinic receptors and activates downstream signalling pathways, inducing cellular proliferation cancer cell lines derived from human pancreas, breast and lung malignancies [48]. In a hamster model of exocrine pancreatic cancer induced by transplacental exposure to ethanol and the tobacco-carcinogen NNK [49], Schuller et al. reported a reduction in the development of pancreatic cancer in offspring who had been given the arachidonic acid pathway inhibitors, ibuprofen or MK886. Tumour development was reduced by 50% or 30%, respectively [49]. The reduction was not a result of an interaction with the drugs on NNK metabolism or cancer initiation events, as the NNK was administered as a single dose on day 15 of gestation, while the preventive treatments started 4 weeks later, when the offspring were weaned from their mothers. Of note, no mutations in the Ki-, N-, or H-ras or p53 genes were found in theses tumours.

There is also recent evidence indicating that NNK is an agonist for nicotinic acetylcholine receptors (nAChRs), and in hamsters, NNK-induced alterations in regulatory nAChRs may contribute to the development of smoking-associated PAC and PDAC by disturbing the balance between cancer-stimulating and inhibiting neurotransmitters [50]. 

In combination, these findings suggest that NNK is a *β*-adrenergic agonist. *β*-adrenergic, AA-dependent regulatory pathways in pancreatic cancer are a possible novel target-dietary or pharmacologic for cancer intervention strategies [51] in an effort towards the prevention and clinical management of pancreatic cancer.

### 3.5. Synergistic Cofactors Contributing to Tobacco Related Pancreatic Carcinogenesis?

While we are beginning to understand some of the key factors involved in pancreatic carcinogenesis, of which smoking is one, the primary etiology of the disease remains poorly understood. Other epidemiological factors may also be important and a genetic predisposition to the disease, supported by reported familial occurrences of the disease [52–54] has long been suspected. As reviewed here, investigators have struggled to show that tobacco contributes directly to the development of acquired mutations in the commonly reported oncogenes and tumour suppressor genes. However, there may well be a more subtle association, with innate or inherited genetic variation, which increases an individuals exposure to damaging agents, or possibly that individuals ability to repair induced DNA damage. There may also be interplay here, as with all polygenic diseases, with other environmental factors. 

Certainly, there is a lot of individual variation in the metabolism of the known tobacco-derived carcinogens. Metabolically activated NNN and NNK bind not only to DNA but also to the protein moiety of haemoglobin resulting in globin-carcinogen adducts. Mass-spectroscopic studies have detected a significant overlap of the globin-carcinogen adduct levels between smokers and nonsmokers, suggesting that individuals vary in their ability to activate TSNAs [55]. As previously mentioned, there is also considerable variation in adduct levels in the pancreas at autopsy among smokers, which could not be accounted for by duration or daily consumption level [30]. While the evidence for a synergistic role between smoking and other factors is limited, the key suspects are mentioned here.

**(a) Dietary Factors**

Various groups of researchers have explored the interaction of tobacco smoke with dietary and other factors in animal models in an attempt to replicate the scenarios found in humans, and there is evidence that some dietary factors act synergistically with these carcinogens. Coadministration of sinigrin (a glucoside found in some plants of the Brassica family such as brussels sprouts, broccoli, and seeds of black mustard) with NNK, for example, resulted in a significant incidence of pancreatic tumors in rats [56]. In a long-term bioassay (24 months) exploring the interaction of dietary fat and tobacco carcinogens, F344 rats were given NNK, in the drinking water. In addition, one group of rats was given a high-corn oil diet and the second group received a low-corn oil diet. There were control groups with similar diet and on plain tap water only. The NNK + high corn oil group gained more weight and developed more number of pancreas tumours as compared to the NNK + low corn oil group. There was no such effect on development of lung cancers in the animals. This elegant and simple bioassay provided supplementary evidence to corroborate epidemiological studies which had suggested a link between daily fat intake and pancreas cancers in smokers [57].

**(b) Obesity**

Obesity is now recognised as a risk factor in development of all cancers, but this is particularly so for pancreatic cancer. Wang et al. reported a 2.6 fold increase in relative risk of a pancreatic cancer associated death in individuals with a body mass index greater than or equal to 35. The animal study above, in which the fatter NNK group fed corn oil developed more pancreatic cancers, supports this although the actual cause is as yet unclear. A further study demonstrated that body mass index was positively correlated to the levels of DNA, lipid peroxidation related adducts and the total aromatic adducts in pancreatic tumour tissues [58]. These observations support the hypothesis that DNA damage related to a combination of carcinogen exposure and lipid peroxidation may be involved in human pancreatic carcinogenesis.

**(c) Alcohol**

A key dietary factor worthy of independent mention is alcohol. Alcohol has long been suspected of contributing to pancreatic cancer development [59, 60], but a synergistic role with tobacco exposure has also been considered. In pregnant animal models, NNK can cross the placenta following tracheal administration and cause its carcinogenic effect-pancreas cancer on the foetus. Concomitant administration of ethanol can greatly potentiated this effect [61], but other than recommending that pregnant women neither drink nor smoke, the human relevance of these findings is unclear. The PANKRAS 1 study investigated the presence of K-Ras mutations in patients who either smoked, or drank alcohol, or did both. The information in this study was taken from patient notes rather than a questionnaire, but reported a higher risk only in those who did one or the other, rather than both [62]. As discussed previously, the frequency of K-Ras mutations is probably not the best tool to assess the impact of suspected carcinogens, and the true impact of smoking in combination with alcohol on pancreatic carcinogenesis has yet to be determined.

**(d) Genetic Factors**

A genetic predisposition is frequently alluded to in reviews of the aetiology of pancreatic cancers [63, 64] and certainly there are genetic disorders, such as Hereditary pancreatitis [65, 66], the Liefraumeni syndrome [67], Peutz-Jeghers syndrome [68] and Hereditary nonpolyposis colon cancer syndrome [69]. Familial atypical melanoma mole syndrome [70] where pancreatic cancer is a major feature. Furthermore, familial pancreatic cancer is a well recognised entity [71–73]. A recent retrospective cross-sectional cases-only study suggested an increased risk for pancreas cancer when multiple environmental risk factors (including tobacco smoke exposure) were present on a background of a family history of pancreas cancer [74]. This study has again emphasised the role of environmental factors in modulating the genetic risk for development of pancreas cancer.

When considering potential synergy between innate susceptibility and tobacco derived carcinogens, we are beginning to look toward genetic variability in the ways in which tobacco derived compounds or metabolites interact with targets, are metabolised, or even the ways in which the damage incurred is repaired. Polymorphisms in genes coding for dopamine receptors and transporters, nicotinic receptors and serotonin receptors, neurotransmitters and transporters [75] may be relevant, and certainly understanding the genetic factors involved in addiction may ultimately result in more effective tobacco cessation programs reducing the incidence of tobacco related diseases, including pancreatic cancer. In addition, however, we are beginning to understand individual variability of susceptibility to damage at a more molecular level.

Both the quantity and duration of exposure to carcinogens may determine adduct formation or the capacity to cause damage. The carcinogens themselves may be metabolised at different rates in individuals. In human pancreatic tissues, there is individual variability in the capacity for, and stereoselectivity of, carbonyl reduction of NNK. In individuals whose microsomes metabolize NNK at a lower rate but form predominantly (S)-NNAL, “stereoselective localization of (S)-NNAL” in the human pancreas might occur, similar to the rat lung, where S-NNAL gets retained resulting in higher incidence of cancers there [76].

One family of proteins with an important role in the metabolism of foreign compounds and drugs, and, therefore, a key suspect in promoting cancer predisposition if relatively ineffective in some circumstances, are the cytochrome P450 (CYP) superfamily. There is a vast literature reporting various genetic polymorphism in CYPs which increase cancer susceptibility. As yet, this is largely unconfirmed, with the exception of CYP2A6 polymorphism and tobacco-induced cancer [77, 78]. The role of these in tobacco related cancer has been summarised by others [78–80] and is not discussed in depth here. In summary, although they have significant effects on tobacco-derived carcinogen metabolism, they are not considered to play an important role in pancreas cancer causation. Phase-2 metabolising enzymes such as Uridine-diphospho-glucuronosyl transferase (UDPGT) [81], Glutathione S-transferase (GST) [82–85] have also been studied but are reported from relatively small single institution studies and need validation in large case-control molecular epidemiological studies.

As well as variability in the capacity to cause damage, those pathways involved in the repair of damage have been studied, with significant individual variation reported. In particular, the role of polymorphisms of genes coding for enzymes and proteins within key metabolic and DNA repair pathways in modulating risk for human pancreas cancer is being investigated and has been the subject of recent reviews [86–88]. Numerous small single centre studies exploring the role of gene-environment interaction and interindividual variability in susceptibility to tobacco derived carcinogen damage [77, 89–95] have recently been reported and require validation. In addition, genome wide association studies (GWAS) in large numbers of individuals with pancreatic cancer are being performed and may ultimately lead us closer to the key genetic factors involved. The first of these confirmed an earlier epidemiologic finding that blood group O results in a decreased risk for pancreas cancer [96]. Another GWAS in Japanese individuals, who have a particularly high risk of pancreatic cancer, was reported in 2010 and identified genes on chromosomes on 6, 7, and 12 [97] and A further similar but larger study has suggested other specific loci on chromosomes 1, 5, and 13 to be associated with an increased risk for the malignancy; however, the functional significance of these findings is not clear.

## 4. Conclusion

Tobacco, as it is used in its most prevalent form, that is, smoking, is the most important risk factor for the development of pancreas cancer. There is compelling epidemiological evidence for a disease-causation effect for this which is backed up by physiological evidence based on laboratory studies on subcellular fractions, cell lines derived from human and animal models of the disease, whole animal models and human studies. There is also significant evidence for interindividual variation in susceptibility to tobacco-derived carcinogens which is a field of intense current research. Understanding of the role of not only tobacco carcinogens, but also the possible toxic role of nicotine itself in humans needs to be emphasised in public awareness campaigns.

Further work is urgently required to explore the complex interactions between genetic, life-style/environmental factors at both laboratory and population levels to understand the disease better and enable improvement in outcome by not only earlier diagnosis, but also innovative and newer modes of intervention.
